# 1-Chloro-1-[(4-chloro­phen­yl)hydrazinyl­idene]propan-2-one

**DOI:** 10.1107/S1600536811026390

**Published:** 2011-07-09

**Authors:** Abdullah M. Asiri, Abdulrahman O. Al-Youbi, Mohie E. M. Zayed, Seik Weng Ng

**Affiliations:** a Chemistry Department, Faculty of Science, King Abdulaziz University, PO Box 80203 Jeddah, Saudi Arabia; b The Center of Excellence for Advanced Materials Research, King Abdul Aziz University, PO Box 8020 Jeddah, Saudi Arabia; c Department of Chemistry, University of Malaya, 50603 Kuala Lumpur, Malaysia

## Abstract

The non-H atoms of the title compound, C_9_H_8_Cl_2_N_2_O, lie nearly on a plane (r.m.s. deviation = 0.110 Å), and the C=N double bond has a *Z* configuration. In the crystal, adjacent mol­ecules are linked by an N—H⋯O_carbon­yl_ hydrogen bond, forming a chain running along [100].

## Related literature

For the synthesis, see: Benincori *et al.* (1990 ▶); Sayed *et al.* (2002 ▶). For background to the title compound, see: Asiri *et al.* (2010 ▶).
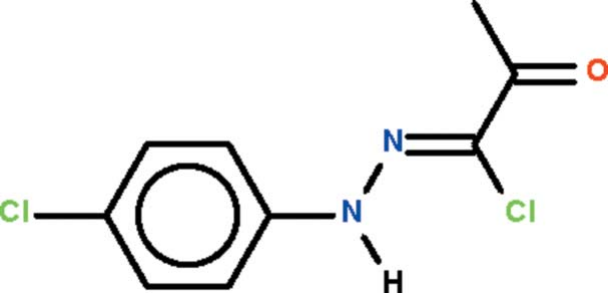


## Experimental

### 

#### Crystal data

C_9_H_8_Cl_2_N_2_O
*M* *_r_* = 231.07
Monoclinic, 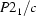

*a* = 5.7558 (4) Å
*b* = 23.3282 (17) Å
*c* = 7.4107 (6) Å
β = 96.976 (7)°
*V* = 987.69 (13) Å^3^
*Z* = 4
Cu *K*α radiation
μ = 5.65 mm^−1^
*T* = 100 K
0.35 × 0.05 × 0.03 mm

#### Data collection

Agilent SuperNova Dual diffractometer with an Atlas detector
Absorption correction: multi-scan (*CrysAlis PRO*; Agilent, 2010 ▶) *T*_min_ = 0.243, *T*_max_ = 0.849
3486 measured reflections
1939 independent reflections
1640 reflections with *I* > 2σ(*I*)
*R*_int_ = 0.036

#### Refinement

*R*[*F* ^2^ > 2σ(*F* ^2^)] = 0.070
*wR*(*F* ^2^) = 0.208
*S* = 1.17
1939 reflections
133 parameters
H atoms treated by a mixture of independent and constrained refinement
Δρ_max_ = 1.28 e Å^−3^
Δρ_min_ = −0.54 e Å^−3^

### 

Data collection: *CrysAlis PRO* (Agilent, 2010 ▶); cell refinement: *CrysAlis PRO*; data reduction: *CrysAlis PRO*; program(s) used to solve structure: *SHELXS97* (Sheldrick, 2008 ▶); program(s) used to refine structure: *SHELXL97* (Sheldrick, 2008 ▶); molecular graphics: *X-SEED* (Barbour, 2001 ▶); software used to prepare material for publication: *publCIF* (Westrip, 2010 ▶).

## Supplementary Material

Crystal structure: contains datablock(s) global, I. DOI: 10.1107/S1600536811026390/xu5260sup1.cif

Structure factors: contains datablock(s) I. DOI: 10.1107/S1600536811026390/xu5260Isup2.hkl

Supplementary material file. DOI: 10.1107/S1600536811026390/xu5260Isup3.cml

Additional supplementary materials:  crystallographic information; 3D view; checkCIF report

## Figures and Tables

**Table 1:** Hydrogen-bond geometry (Å, °)

*D*—H⋯*A*	*D*—H	H⋯*A*	*D*⋯*A*	*D*—H⋯*A*
N2—H2⋯O1^i^	0.85 (8)	2.26 (8)	3.029 (6)	150 (7)

Symmetry code: (i) 
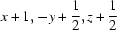
.

**Fig. 1.**
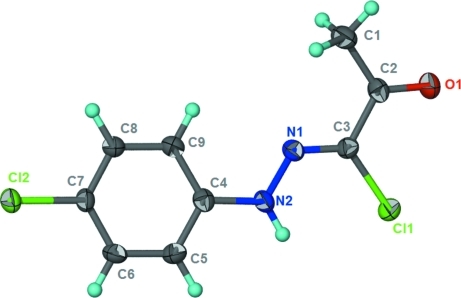
Thermal ellipsoid plot (Barbour, 2001) of C9H8Cl2N2O at the 70% probability level; hydrogen atoms are drawn as spheres of arbitrary radius.

## supplementary crystallographic information

### Comment

We have previously reported the synthesis of ethyl (*Z*)-2-chloro-2-(2-phenylhydrazin-1-ylidene) acetate by the reaction of benzenediazonium chloride with ethyl 2-chloro-3-oxobutanoate (Asiri *et al.*, 2010). The compound is an ester. In the present study, the use of a substituted benzenediazonium chloride and the methyl ester (instead of the ethyl ester) afforded a 1-chloro-1-(arylhydrazono)-2-propanone. Such ketones are intermediates in the synthesis of pyrazoles (Sayed *et al.*, 2002) and other heterocycles (Benincori *et al.*, 1990). In the 4-chloro substituted compound (Scheme I, Fig. 1), the non-hydrogen atoms lie on a plane [r.m.s. deviation 0.110 Å] (Scheme I, Fig. 1). The C_aryl_–N(H)–N═ C(S)═O portion adopts an extended zigzag conformation. Adjacent molecules are linked by an *N*–*H*···O_carbonyl_ hydrogen bond to form a chain running [1 0 0].

### Experimental

To a stirred solution of methyl 2-chloro-3-oxobutanoate (1.64 g, 10 mmol) in ethanol (100 ml) was added sodium acetate trihydrate (1.30 g, 10 mmol). The mixture was chilled to 273 K and then treated with a cold solution of *p*-nitrobenzenediazonium chloride, prepared by diazotizing *p*-chloroaniline (1.20 g, 10 mmol) dissolved in 6*M* hydrochloric acid (6 ml) with a solution of sodium nitrite (0.70 g, 10 mmol) in water (10 ml). The addition of the diazonium salt solution was carried out with rapid stirring over a period of 20 min. The reaction mixture was stirred for further 15 min. and left for 3 h in refrigerator. The resulting solid was collected by filtration and washed thoroughly with water. The crude product was crystallized from ethanol to give the corresponding hydrazonoyl chloride.

### Refinement

Carbon-bound H-atoms were placed in calculated positions [C—H 0.95 to 0.98 Å, *U*_iso_(H) 1.2 to 1.5*U*_eq_(C)] and were included in the refinement in the riding model approximation.

The amino H-atom was located in a difference Fourier map, and was freely refined.

The final difference Fourier map had a peak in the vicinity of H1*b*.

Omitted from the refinement were (-3 5 1), (-3 13 1), (-3 4 2) and (-3 3 3).

### Crystal data

**Table:**

C_9_H_8_Cl_2_N_2_O	*F*(000) = 472
*M**_r_* = 231.07	*D*_x_ = 1.554 Mg m^−^^3^
Monoclinic, *P*2_1_/*c*	Cu *K*α radiation, λ = 1.54184 Å
Hall symbol: -P 2ybc	Cell parameters from 1477 reflections
*a* = 5.7558 (4) Å	θ = 3.8–74.1°
*b* = 23.3282 (17) Å	µ = 5.65 mm^−^^1^
*c* = 7.4107 (6) Å	*T* = 100 K
β = 96.976 (7)°	Prism, yellow
*V* = 987.69 (13) Å^3^	0.35 × 0.05 × 0.03 mm
*Z* = 4	

### Data collection

**Table:**

Agilent SuperNova Dual diffractometer with an Atlas detector	1939 independent reflections
Radiation source: SuperNova (Cu) X-ray Source	1640 reflections with *I* > 2σ(*I*)
Mirror	*R*_int_ = 0.036
Detector resolution: 10.4041 pixels mm^-1^	θ_max_ = 74.3°, θ_min_ = 3.8°
ω scans	*h* = −6→7
Absorption correction: multi-scan (*CrysAlis PRO*; Agilent, 2010)	*k* = −27→28
*T*_min_ = 0.243, *T*_max_ = 0.849	*l* = −9→9
3486 measured reflections	

### Refinement

**Table:**

Refinement on *F*^2^	Secondary atom site location: difference Fourier map
Least-squares matrix: full	Hydrogen site location: inferred from neighbouring sites
*R*[*F*^2^ > 2σ(*F*^2^)] = 0.070	H atoms treated by a mixture of independent and constrained refinement
*wR*(*F*^2^) = 0.208	*w* = 1/[σ^2^(*F*_o_^2^) + (0.0903*P*)^2^ + 4.1934*P*] where *P* = (*F*_o_^2^ + 2*F*_c_^2^)/3
*S* = 1.17	(Δ/σ)_max_ = 0.002
1939 reflections	Δρ_max_ = 1.28 e Å^−^^3^
133 parameters	Δρ_min_ = −0.54 e Å^−^^3^
0 restraints	Extinction correction: *SHELXL97* (Sheldrick, 2008), Fc^*^=kFc[1+0.001xFc^2^λ^3^/sin(2θ)]^-1/4^
Primary atom site location: structure-invariant direct methods	Extinction coefficient: 0.0015 (7)

### Special details

**Table:**

Geometry. All e.s.d.'s (except the e.s.d. in the dihedral angle between two l.s. planes) are estimated using the full covariance matrix. The cell e.s.d.'s are taken into account individually in the estimation of e.s.d.'s in distances, angles and torsion angles; correlations between e.s.d.'s in cell parameters are only used when they are defined by crystal symmetry. An approximate (isotropic) treatment of cell e.s.d.'s is used for estimating e.s.d.'s involving l.s. planes.

### Fractional atomic coordinates and isotropic or equivalent isotropic displacement parameters (Å^2^)

**Table:**

	*x*	*y*	*z*	*U*_iso_*/*U*_eq_	
Cl1	0.2378 (2)	0.21916 (5)	0.50652 (16)	0.0233 (4)	
Cl2	0.7056 (2)	0.56866 (5)	0.85227 (16)	0.0253 (4)	
O1	−0.2165 (6)	0.23416 (15)	0.2828 (5)	0.0272 (8)	
N1	0.1754 (7)	0.33248 (18)	0.5054 (5)	0.0194 (9)	
N2	0.3787 (7)	0.33782 (18)	0.6093 (5)	0.0204 (9)	
H2	0.464 (14)	0.308 (3)	0.632 (10)	0.05 (2)*	
C1	−0.2428 (9)	0.3362 (2)	0.2694 (8)	0.0290 (12)	
H1A	−0.3553	0.3296	0.1612	0.044*	
H1B	−0.1229	0.3634	0.2402	0.044*	
H1C	−0.3244	0.3520	0.3669	0.044*	
C2	−0.1284 (9)	0.2803 (2)	0.3310 (7)	0.0215 (10)	
C3	0.0947 (9)	0.2835 (2)	0.4510 (6)	0.0193 (10)	
C4	0.4510 (8)	0.3930 (2)	0.6690 (6)	0.0197 (10)	
C5	0.6761 (9)	0.4003 (2)	0.7582 (7)	0.0235 (11)	
H5	0.7773	0.3682	0.7794	0.028*	
C6	0.7533 (9)	0.4543 (2)	0.8162 (6)	0.0226 (10)	
H6	0.9070	0.4595	0.8768	0.027*	
C7	0.6036 (9)	0.5004 (2)	0.7845 (6)	0.0195 (10)	
C8	0.3792 (9)	0.4940 (2)	0.6972 (7)	0.0221 (10)	
H8	0.2789	0.5263	0.6771	0.027*	
C9	0.3016 (8)	0.4404 (2)	0.6392 (6)	0.0209 (10)	
H9	0.1474	0.4356	0.5793	0.025*	

### Atomic displacement parameters (Å^2^)

**Table:**

	*U*^11^	*U*^22^	*U*^33^	*U*^12^	*U*^13^	*U*^23^
Cl1	0.0260 (7)	0.0181 (6)	0.0248 (6)	0.0025 (4)	−0.0012 (5)	0.0005 (4)
Cl2	0.0281 (7)	0.0178 (6)	0.0279 (6)	−0.0023 (5)	−0.0050 (5)	−0.0019 (4)
O1	0.0274 (19)	0.0194 (18)	0.033 (2)	−0.0044 (15)	−0.0023 (15)	−0.0029 (15)
N1	0.019 (2)	0.021 (2)	0.0179 (18)	−0.0010 (16)	−0.0002 (15)	0.0004 (16)
N2	0.021 (2)	0.018 (2)	0.021 (2)	−0.0031 (17)	−0.0028 (16)	−0.0005 (16)
C1	0.024 (3)	0.025 (3)	0.036 (3)	−0.003 (2)	−0.007 (2)	0.002 (2)
C2	0.021 (2)	0.020 (2)	0.024 (2)	−0.0049 (19)	0.0022 (19)	−0.0011 (19)
C3	0.021 (2)	0.018 (2)	0.019 (2)	0.0010 (18)	0.0013 (18)	0.0013 (18)
C4	0.024 (2)	0.020 (2)	0.016 (2)	−0.0012 (19)	0.0014 (18)	0.0006 (18)
C5	0.022 (2)	0.023 (3)	0.024 (2)	0.005 (2)	−0.0008 (19)	0.000 (2)
C6	0.018 (2)	0.027 (3)	0.021 (2)	0.003 (2)	−0.0032 (18)	−0.001 (2)
C7	0.024 (2)	0.015 (2)	0.019 (2)	−0.0017 (18)	−0.0001 (18)	−0.0013 (17)
C8	0.020 (2)	0.020 (2)	0.025 (2)	0.0015 (19)	−0.0023 (19)	−0.0008 (19)
C9	0.018 (2)	0.023 (2)	0.021 (2)	0.0008 (19)	−0.0002 (18)	−0.0001 (19)

### Geometric parameters (Å, °)

**Table:**

Cl1—C3	1.737 (5)	C2—C3	1.472 (7)
Cl2—C7	1.749 (5)	C4—C5	1.392 (7)
O1—C2	1.225 (6)	C4—C9	1.401 (7)
N1—C3	1.280 (6)	C5—C6	1.386 (7)
N1—N2	1.326 (6)	C5—H5	0.9500
N2—C4	1.407 (6)	C6—C7	1.382 (7)
N2—H2	0.85 (8)	C6—H6	0.9500
C1—C2	1.506 (7)	C7—C8	1.381 (7)
C1—H1A	0.9800	C8—C9	1.379 (7)
C1—H1B	0.9800	C8—H8	0.9500
C1—H1C	0.9800	C9—H9	0.9500
			
C3—N1—N2	121.8 (4)	C5—C4—N2	119.0 (4)
N1—N2—C4	118.4 (4)	C9—C4—N2	121.3 (4)
N1—N2—H2	119 (5)	C6—C5—C4	120.2 (5)
C4—N2—H2	123 (5)	C6—C5—H5	119.9
C2—C1—H1A	109.5	C4—C5—H5	119.9
C2—C1—H1B	109.5	C7—C6—C5	119.1 (5)
H1A—C1—H1B	109.5	C7—C6—H6	120.5
C2—C1—H1C	109.5	C5—C6—H6	120.5
H1A—C1—H1C	109.5	C6—C7—C8	121.5 (4)
H1B—C1—H1C	109.5	C6—C7—Cl2	118.7 (4)
O1—C2—C3	121.3 (5)	C8—C7—Cl2	119.8 (4)
O1—C2—C1	121.5 (4)	C9—C8—C7	119.6 (5)
C3—C2—C1	117.2 (4)	C9—C8—H8	120.2
N1—C3—C2	119.4 (4)	C7—C8—H8	120.2
N1—C3—Cl1	123.6 (4)	C8—C9—C4	119.8 (4)
C2—C3—Cl1	117.0 (4)	C8—C9—H9	120.1
C5—C4—C9	119.7 (5)	C4—C9—H9	120.1
			
C3—N1—N2—C4	−177.6 (4)	N2—C4—C5—C6	179.0 (4)
N2—N1—C3—C2	−178.0 (4)	C4—C5—C6—C7	0.1 (8)
N2—N1—C3—Cl1	0.4 (7)	C5—C6—C7—C8	0.2 (8)
O1—C2—C3—N1	−176.7 (5)	C5—C6—C7—Cl2	−178.2 (4)
C1—C2—C3—N1	3.6 (7)	C6—C7—C8—C9	−0.2 (8)
O1—C2—C3—Cl1	4.8 (7)	Cl2—C7—C8—C9	178.1 (4)
C1—C2—C3—Cl1	−174.9 (4)	C7—C8—C9—C4	−0.1 (7)
N1—N2—C4—C5	−171.9 (4)	C5—C4—C9—C8	0.5 (7)
N1—N2—C4—C9	7.6 (7)	N2—C4—C9—C8	−179.0 (4)
C9—C4—C5—C6	−0.5 (7)		

### Hydrogen-bond geometry (Å, °)

**Table:**

*D*—H···*A*	*D*—H	H···*A*	*D*···*A*	*D*—H···*A*
N2—H2···O1^i^	0.85 (8)	2.26 (8)	3.029 (6)	150 (7)

Symmetry codes: (i) *x*+1, −*y*+1/2, *z*+1/2.
